# Cloud computing approaches for prediction of ligand binding poses and pathways

**DOI:** 10.1038/srep07918

**Published:** 2015-01-22

**Authors:** Morgan Lawrenz, Diwakar Shukla, Vijay S. Pande

**Affiliations:** 1 Department of Chemistry, Stanford University, Stanford, CA 94305; 2 SIMBIOS NIH Center for Biomedical Computation, Stanford University, Stanford, CA 94305

## Abstract

We describe an innovative protocol for *ab initio* prediction of ligand crystallographic binding poses and highly effective analysis of large datasets generated for protein-ligand dynamics. We include a procedure for setup and performance of distributed molecular dynamics simulations on cloud computing architectures, a model for efficient analysis of simulation data, and a metric for evaluation of model convergence. We give accurate binding pose predictions for five ligands ranging in affinity from 7 nM to > 200 *μ*M for the immunophilin protein FKBP12, for expedited results in cases where experimental structures are difficult to produce. Our approach goes beyond single, low energy ligand poses to give quantitative kinetic information that can inform protein engineering and ligand design.

Structural information is crucial for understanding functional mechanisms of ligands that bind proteins to modulate biochemical signals. In particular, rational drug design requires accurate information about ligand binding poses within the protein. X-ray crystallography gives the most detailed experimental structural information, but can be difficult to produce, particularly for membrane-bound protein-ligand complexes^1^ or for low affinity ligands^2^. Molecular dynamics (MD) simulations use physically rigorous models of protein, ligand, and solution to give an atomistic and dynamic description of ligand binding and significantly inform drug design without the inaccuracies that plague simplified approaches^3^,^4^. Ligand binding events have been captured using long timescale MD simulations performed on specialized hardware^5^,^6^ and for only one ligand to a protein^7^,^8^. We adapt an approach that has been successful in studies of protein folding^9^ and conformational change^10^,^11^ in which we build a statistical Markov state model (MSM) from ligand binding MD simulations performed on cloud computing architectures. Our model gives accurate *ab initio* predictions of ligand binding poses for five ligands labeled as L2, L3, L6, L9, and 5 Androstan-3α-ol in Fig. 1, with a range of binding affinities for the FK506 binding protein 12 (FKBP12), a class of immunophilins with peptidyl-prolyl isomerase (PPI) activity^12^ and diverse roles in cellular signaling, particularly in immunosuppression^13^ and neurological function^14^,^15^. Furthermore, our approach allows a kinetic description of the binding mechanism, including association rates, protein-ligand encounter complexes, secondary binding sites or “hot spots”, and druggable cryptic sites on the protein^16^. A summary of the steps involved in our approach is illustrated in Fig. 2.

## Results

### MSM-guided scheme for improved sampling of ligand binding events

To generate data for our model, we set up an initial round of MD simulations with diverse starting structures for the ligand and protein. These starting structures include unbound states, near-bound states, and initial predicted bound states from small molecule docking to the FK506 binding site. With predicted near-bound states, we aim to enhance the sampling of binding events by incorporating available experimental information, which can include binding residues identified from structural or mutagenic studies. We drive the ligand binding MD simulations with statistical MSMs, which are constructed by first clustering the aggregate simulation data by the protein-aligned positions of the ligand, using root mean squared deviation (RMSD) as a metric. Then, transitions between these structurally defined states are counted from the raw trajectory data in order to build a transition matrix at a particular time unit that lumps the clusters into Markovian states, such that intra-state transitions are faster than inter-state transitions (see Methods). We run new simulations using an adaptive sampling approach that has been shown to improve conformational sampling by seeding new simulations based on existing MSM states^17^. For this study, we launched three adaptive sampling rounds, detailed in Supplementary Table 1, with the initial round performed on local computing clusters at Stanford and subsequent rounds on the distributed computing network Folding@home^18^. The number of adaptive sampling rounds can be chosen based on availability of resources as well as convergence plots, described below. Our results illustrate the power of MSMs, which can stitch together events that occur to different extents in many independent, parallel simulations, but can take a long wall clock time to occur altogether in any single simulation. For example, in seven long timescale simulations performed in Shan, *et al*^6^, only 3 binding events for dasatinib to Src kinase were observed in 115 *μ*s, while we observe hundreds of binding events for our ligands (Supplementary Table 1).

### MSM-derived equilibrium populations find accurate ligand binding poses and druggable binding sites

**Figure 1:**
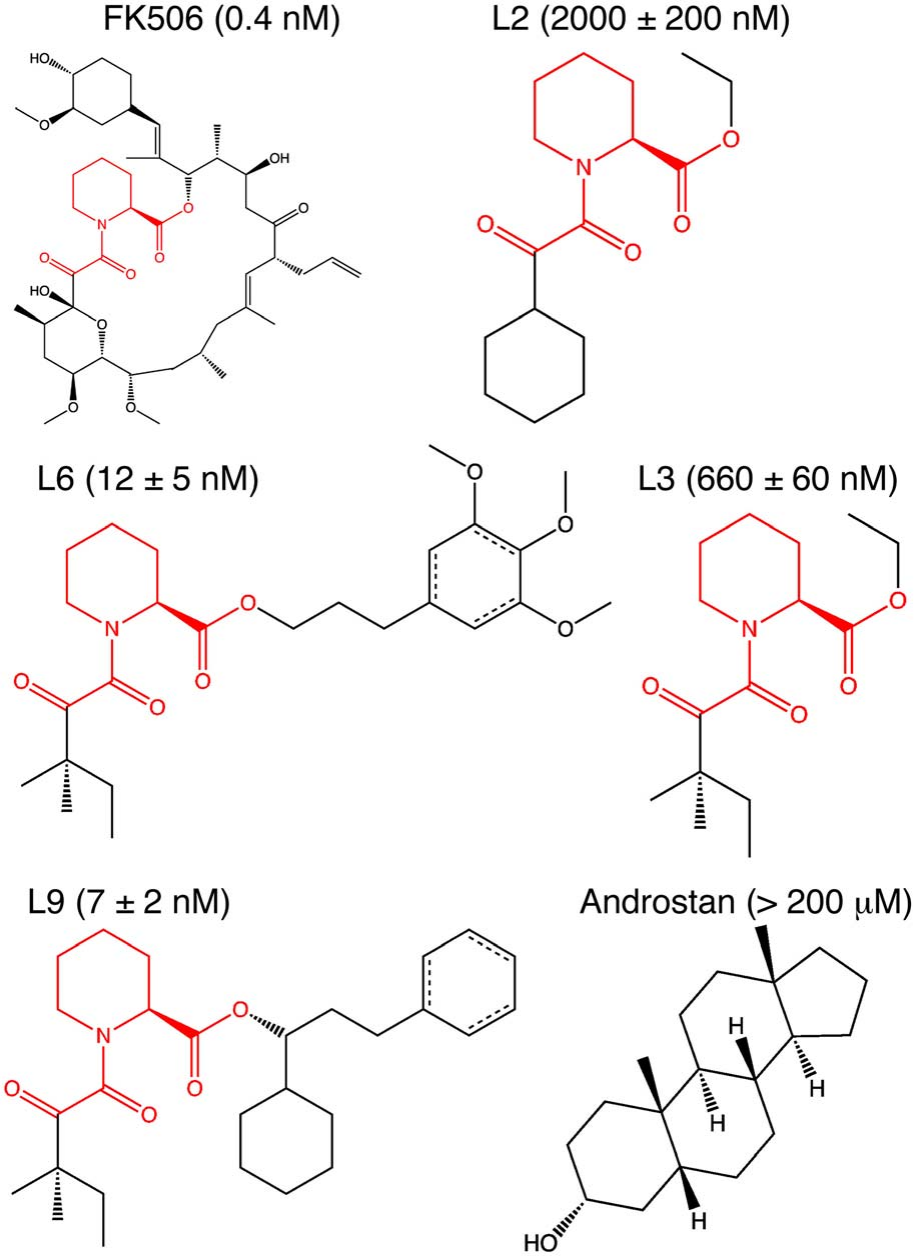
FKBP12 ligands used for predictions in this study. The chemical structures highlight the common core in the ligands L2, L3, L6 and L9 derived from FK506 from Holt, *et al.* Ki values are also listed^19^,^43^. A structure is available for L9^43^, but structure factors for computing electron densities are only available for FK506^25^.

**Figure 2:**
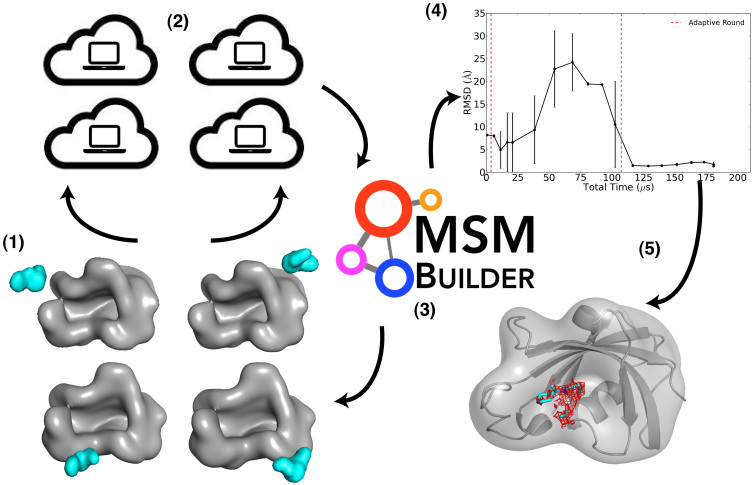
Overview of the scheme used to for FKBP12 ligand predictions. The scheme for the approach described in this study is illustrated, with steps as follows: 1) select diverse structures for the protein-ligand complex, (2) perform extensive MD simulations on cloud computing architectures, (3) construct a ligand-binding MSM at the end of a simulation period, (4) evaluate the MSM convergence, and repeat steps 1–4 until convergence is reached and reliable predictions are available (5). In step 4, we show the convergence of the lowest free energy state for L2, selected from MSMs built at ≈ 10 *μ*s intervals and plotted as the state RMSD to the reference pose as the rolling mean with standard deviations over 2 data points (≈20 *μ*s).

**Figure 3:**
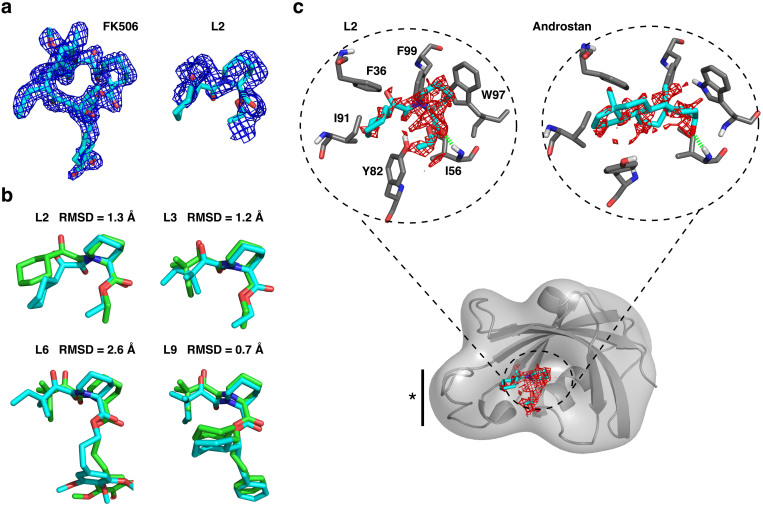
Comparison of FKBP12 ligand pose predictions with experiment. (a) The available electron density for FK506^25^ is shown in the left panel, compared with the adjusted density that corresponds to the common scaffold for L2, in the right panel. The density is shown at the 1*σ* contour level of the 2Fo–Fc difference map computed in PHENIX^44^. The predicted L2 pose is shown in cyan stick representation to illustrate overlap with the available density. (b). Overlap of the predicted pose from the MSM (cyan), determined as the converged, highly populated ligand MSM state, with the validation pose (green). The validation pose is derived from crystallography experiments for L9^43^, or from overlap and minimization of common scaffolds for L2, L3, and L6 with the L9 and FK506 structures, as done previously for accurate binding free energy predictions for these ligands^28^,^41^. The RMSD between the structures is listed. (c). The 1.0 kcal/mol contour of the 3-D MSM-weighted free energy map within the active site is shown for L2 and 5 Androstan-3α-ol, with key binding residues labeled and a solid bar with a star denoting the 80's loop region. The free energy minimum surface is shown for all ligands in Supplementary Information Fig. 3. The L2 surface can be closely compared to the electron density contour in (a).

We build MSMs from simulation data at approximately 10 *μ*s intervals and analyze metastable ligand states that can be ranked according to the maximum likelihood estimate of the equilibrium population derived from the MSM transition matrix. The final rankings are listed for the FKBP12 ligands in Supplementary Table 2. The top populated pose is monitored over time for convergence behavior, plotted as the pose RMSD to the reference structures available for the FK506-derived ligands, described in Fig. 1. In the case of the blind prediction for 5 Androstan-3α-ol, the RMSD to the final predicted pose, or distances to known key binding residues, is evaluated for convergence. The convergence plot is shown for L2 in the scheme of Fig. 2, and shown for all ligands in Supplementary Fig. 1. These plots help us to evaluate our confidence in the predictions and guide the iterative process of adaptive sampling. The ligand poses converge to < 3 Å RMSD to the available experimental structure after ≈ 100 *μ*s of aggregate simulation time. Our final predictions for the FK506-derived ligands have close overlap with the FK506-derived electron density, shown for L2 in Fig. 3a, as well as with the reference poses in Fig. 3b, which are in agreement at < 1.3 Å for three out of four ligands.

The entire converged MSM can be incorporated into a 3-D free energy map (see Methods) that reveals both low and high free energy ligand binding sites on the protein. The minimal free energy surface in the active site of FKBP12 is illustrated in Fig. 3c for L2 and 5 Androstan-3α-ol, and shown for all ligands in Supplementary Fig. 2. These surfaces are strikingly similar to the FK506-derived electron densities (observed for L2 in Fig. 3a, and for all ligands in Supplementary Fig. 2). The surfaces also overlap very well with the correctly predicted binding pose, shown in cyan stick representation. The similarity of the predicted free energy surfaces and experimental electron densities demonstrates the utility of our approach for ascribing atomistic detail to complement, or use in place of, difficult crystallography experiments. We see that similar interactions are maintained with FKBP12 in the binding poses for L2 and the blindly predicted 5 Androstan-3α-ol in Fig. 3c. Four of these interactions, hydrophobic contacts with F36, I91, and F99, as well as a hydrogen bond to I56, are experimentally validated binding residues for this steroidal class^19^.

In addition to prediction of the lowest free energy binding pose, the MSM free energy map can be contoured to identify other druggable sites on the protein and guide structure-activity relationship experiments. Low free energy surfaces extend into regions beyond the top population binding pose, revealing un-utilized but favorable regions of the binding site that could be targeted with new chemical moieties or incorporated in the design of novel ligand scaffolds. Our results directly support this application. The low free energy surface of the MSM 3-D free energy map, illustrated in Supplementary Fig. 3 for L2, allows identification of the region occupied by ring groups in L6, L9, and FK506, which contributes an approximately two orders of magnitude increase in binding affinity. Scaling the free energy surface to high free energy surfaces reveals lower probability sites, which were found in this study to be nonspecific binding sites, labeled A, B, and C in Supplementary Fig. 4 and are visited by all ligands in the simulations. In other proteins, these surfaces could identify druggable cryptic or allosteric binding sites.

### Characterization of binding pathways using kinetic information from the MSM

We also gain unbiased kinetic information about the binding process from the MSM. Calculated association rates for the ligands, listed in Supplementary Table 3, have a favorable ballpark comparison with the available experimental information for FKBP12 kinetics. Using transition path theory^20^,^21^, we find high probability binding pathways, which provide an atomistic description of predominant encounter complexes and ligand transition states. The FKBP12 ligand binding pathways are characterized by the encounter complex in Supplementary Table 4, with many pathways proceeding through the nonspecific sites A, B, and C, as well as through metastable conformations near the C- and N-terminal portions of the 80's loop. This loop region is labeled with a star in Fig. 3c and has been experimentally shown to mediate binding of FK506 as well as other FKBP signaling partners^22^. We also characterize intermediate ligand transition states; the FK506-derived ligands form a common flipped state, shown for L2 and L6 in Supplementary Fig. 5. Key binding residues I56 and Y82 participate in a hydrogen bond exchange with ligand carbonyl groups to convert the ligand to the fully bound state. This transition requires rotation about the proline-mimetic *ϕ* and *ψ* angles of the ligands and corresponds to the FKBP12 enzymatic conversion of proline from a trans to cis conformation^23^. Our analysis of the FKBP12 ligand binding pathways have biochemical validation and allow testable predictions for optimizing binding kinetics via ligand changes, in the case of drug design, or protein mutations, in the case of protein engineering.

## Discussion

Altogether, we provide a well-defined protocol for incorporating receptor and ligand flexibility into a mechanistic or drug design study, particularly when structural information about the system of interest may be difficult to obtain or incomplete. Until now, ligand binding events have only been captured with very long timescale simulations. Our MSMs aggregate information from simulations on diverse computing architectures, which is ideal for data generated from growing cloud computing resources. As computing power advances, we can more quickly and easily create accurate models of biological interactions and produce extensive datasets that inform predictions. Our approach aims to improve efficiency of the generation of “Big Data” on protein-ligand dynamics and to create a human-readable view of ligand binding from data analysis. The goal is to increase the interface between theory and experiment, for understanding biological mechanisms and improving efficiency of structure-based drug design.

## Methods

### Building the initial protein-ligand ensembles

To create predicted near-bound starting states, small molecule docking was performed using the program Surflex^24^, and the crystal structure of FK506-bound FKBP12^25^ to identify and target the FKBP active site. Information about the binding poses of the specific ligands in this study was not used. The option pgeomx was employed to dock each protonated (pH = 7) ligand to a protomol, which is the inverse 3-D representation of the binding site generated by Surflex from the FK506-bound FKBP12 structure. The top 20 binding poses from the docking were used as initial predicted bound states. Near bound and unbound states were generated by translating these poses in a 20 Å grid around the binding site using the program VMD, removing poses that produced steric clashes with the protein when preparing the complex with AMBER12 leap (see below). These configurations were used to initialize ≈ 50 independent trajectories on local Stanford clusters (Supplementary Table 1). Further simulations were launched according to an adaptive sampling scheme, described below.

### Setup and performance of MD simulations

Molecular Dynamics (MD) simulations were performed using GROMACS 4.5^26^ on CPU resources from both local Stanford clusters and the Folding@home distributed computing platform. The protein-ligand complexes were set up in AMBER12 leap using the AMBER99SB-ILDN parameters^27^ and ligands were parametrized as in a previous work^28^ using the Generalized Amber Force Field (GAFF)^29^. All parameters were ported to GROMACS with the program acpype^30^. The complexes were solvated using GROMACS tools in a triclinic solvent box with dimensions 68 · 68 · 48 Å^3^ with ≈ 7000 TIP3P^31^ water molecules such that water extended at least 10 Å away from the surface of the protein. Four chloride ions were added to neutralize the charge. For performing simulations, GROMACS input files were edited such that covalent bonds involving hydrogen atoms were constrained with LINCS^32^ and particle mesh Ewald^33^ with cubic interpolation and a 1.2 Å grid spacing for Fast Fourier Transform was used to treat long-range electrostatic interactions. The neighbor list was updated with a grid search using the switching algorithm with a van-der-Waals cutoff of 9 Å and short-range neighbor list and electrostatic cutoffs of 10 Å. The starting structures were initially minimized for 50,000 steps with steepest descent and equilibrated for 200 ps in the NVT ensemble, then equilibrated for 5 ns in the NPT ensemble before production NPT ensemble simulations at 300 K with a Nose-Hoover thermostat^34^ and constant pressure at 1 atm respectively, with semiisotropic coupling to a Parrinello-Rahman barostat^35^ with a time constant of 5 ps and compressibility of 4.5 · 10^−5^ per bar. Periodic boundary conditions were used for all simulations and randomized starting velocities to initialize the simulations were assigned from a Maxwell-Boltzmann distribution.

### Building Markov state models

All MSMs were built by first clustering the simulation data, at an interval of 10 ns in the final MSM down to 100 ps intervals for MSMs with less data (Supplementary Table 5). We used a k-centers clustering algorithm, followed by the hybrid k-medioids algorithm in MSMBuilder2^36^ with the metric of root mean square deviation (RMSD) of protein backbone aligned ligand coordinates, with a cutoff of 3 Å for cluster similarity. The simulation data was then assigned to these clusters and, in MSMBuilder2, used to construct a transition count matrix *Cij*, the number of observed transitions from state *i* at time *t* to state *j* at time *t* + *τ*, where *τ* is the lag time of the model and corresponding transition probability matrix *Pij*, the probability of transitioning from state *i* at time *t* to state *j* at time *t* + *τ*. The Markov lag time *τ* is the smallest time interval in which the data can be demonstrated as Markovian and was determined by plotting rates *k* from eigenvalues *μ* of the transition probability matrix at varied lag times *τ* as 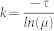. This equation comes from the equivalence between discrete time MSMs and continuous time master equation^37^,^38^,^39^. These rates, called implied timescales, should be unchanged when a system is Markovian, to satisfy the Chapman-Kolmogorov test^40^, and were monitored for all models. If a model for a particular aggregate simulation time did not indicate this behavior, it was discarded and more simulation data was added. The implied timescales for the final MSM are shown in Supplementary Fig. 6. Lag times varied among the datasets and are listed in Supplementary Table 6, with *τ* = 10 ns used for all ligands in the final MSM.

### Adaptive Sampling

Adaptive sampling was performed first on local computing clusters at Stanford and subsequent rounds on the distributed computing network Folding@home^18^. New rounds were launched after monitoring the convergence plots in Supplementary Fig. 1, and are detailed in Supplementary Table 1, along with the total binding and unbinding events observed for the final dataset. Convergence behavior of the RMSD of the top populated ligand MSM state with respect to a reference structure at 10 *μ*s intervals of aggregate simulation time, was not seen until the third round of adaptive sampling. Experimentally derived reference structures were available for the FK506-derived ligands, but distance to binding residues can be used for ligands without experimental information. The reference structure corresponds to the available crystal structure for L9, and is generated by overlap and minimization of the common scaffolds for L2, L3, and L6 with the L9 and FK506 structures, as done previously for accurate binding free energy predictions for these ligands^28^,^41^.

### Mapping the Markov model derived free energies to 3-D space

The final ligand MSM equilibrium state probabilities were mapped to a 1 Å grid space centered on the FKBP protein using a conditional probability assignment for each 1 Å^3^ grid cell *c_i_*. Occupancy of each cubic cell of the grid by the ligand in a Markov state *m_j_* is evaluated, with an occupancy of 1 assigned to the cell if any heavy atom of the ligand is found in the cell, given its conformation relative to the protein in the Markov state *m_j_*. Then, the MSM-derived equilibrium probabilities *P*(*m_j_*) are mapped onto the occupied cell as 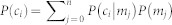. The probabilities were converted to free energies as *k_B_Tln*(*P*(*c_i_*)) and the free energy minimum was set to zero by subtracting the minimal value from all free energy data. This 3-D map was converted to OpenDX format for easy visualization of isosurfaces in VMD^42^. The code for converting the MSM into the 3-D map is provided at https://github.com/mlawrenz/LigandPMF3D.git and is compatible with MSMBuilder2 output.

### Transition path theory

The implementation of transition path theory^20^,^21^ in MSMBuilder2 was used to trace high flux paths between unbound ligand states, defined as ligand positions > 20 Å from any protein surface atom, to bound ligand states with ligand RMSD < 3 Å to the predicted crystallographic pose. Characterized pathways were those with at least 50% of the maximum flux pathway. Association rates were computed as the average mean first passage time (MFPT) from unbound to bound states, defined with the same criteria.

## Author Contributions

V.S.P. conceived and supervised the project. M.L. designed and set up all simulations, processed and analyzed all data, and wrote the paper with input from D.S. and V.S.P. The simulations on the Folding@home network were managed by D.S.

## Supplementary Material

Supplementary Information Supplementary information for cloud computing approaches for prediction of ligand binding poses and pathways

## References

[b1] Chae P. S. *et al.* Maltose-neopentyl glycol (MNG) amphiphiles for solubilization, stabilization and crystallization of membrane proteins. Nat. Methods 7, 1003–1008 (2010). 21037590 10.1038/nmeth.1526 PMC3063152

[b2] Hoeppner A., Schmitt L. & Smits S. Proteins and their ligands: Their importance and how to crystallize them. Advanced Topics on Crystal Growth Ferreira, S. O. (ed.) (InTech, 2013).

[b3] Feixas F., Lindert S., Sinko W. & McCammon J. A. Exploring the role of receptor flexibility in structure-based drug discovery. Biophys. Chem. 186, 31–45 (2014). 24332165 10.1016/j.bpc.2013.10.007 PMC4459653

[b4] Schneider G. Virtual screening: an endless staircase? Nat. Rev. Drug Discovery 9, 273–276 (2010). 10.1038/nrd3139 20357802

[b5] Dror R. O. *et al.* Activation mechanism of the 2-adrenergic receptor. Proc. Natl. Acad. Sci. U. S. A. 108, 18684–18689 (2011). 22031696 10.1073/pnas.1110499108 PMC3219117

[b6] Shan Y. *et al.* How does a drug molecule find its target binding site? J. Am. Chem. Soc. 133, 9181–9183 (2011). 21545110 10.1021/ja202726y PMC3221467

[b7] Buch I. & Giorgino T. Complete reconstruction of an enzyme-inhibitor binding process by molecular dynamics simulations. *Proc*. Natl. Acad. Sci. U. S. A 108, 10184–10189 (2011). 10.1073/pnas.1103547108 PMC3121846 21646537

[b8] Silva D.-A., Bowman G. R., Sosa-Peinado A. & Huang X. A Role for Both Conformational Selection and Induced Fit in Ligand Binding by the LAO Protein. PLoS Comput. Biol. 7, e1002054 (2011). 21637799 10.1371/journal.pcbi.1002054 PMC3102756

[b9] Voelz V. A., Bowman G. R., Beauchamp K. & Pande V. S. Molecular simulation of ab initio protein folding for a millisecond folder NTL9(1–39). J. Am. Chem. Soc. 132, 1526–1528 (2010). 20070076 10.1021/ja9090353 PMC2835335

[b10] Kohlhoff K. J. *et al.* Cloud-based simulations on Google Exacycle reveal ligand modulation of GPCR activation pathways. Nature Chem. 6, 15–21 (2013). 24345941 10.1038/nchem.1821 PMC3923464

[b11] Shukla D., Meng Y., Roux B. & Pande V. S. Activation pathway of Src kinase reveals intermediate states as targets for drug design. Nature Comm. 5, 3397 (2014). 10.1038/ncomms4397 PMC4465921 24584478

[b12] Chen Y. G., Liu F. & Massague J. Mechanism of TGF receptor inhibition by FKBP12. EMBO J. 16, 3866–387 (1997). 9233797 10.1093/emboj/16.13.3866 PMC1170011

[b13] Griffith J. P. *et al.* X-ray structure of calcineurin inhibited by the immunophilin-immunosuppressant FKBP12-FK506 complex. Cell 82, 507–522 (1995). 7543369 10.1016/0092-8674(95)90439-5

[b14] Chong Z. Z., Shang Y. C., Zhang L. & Wang S. Mammalian target of rapamycin: hitting the bull's-eye for neurological disorders. Oxid. Med. Cell Longev. 3, 374–391 (2010). 21307646 10.4161/oxim.3.6.14787 PMC3154047

[b15] Sugata H., Matsuo K., Nakagawa T. & Takahashi M. A peptidyl–prolyl isomerase, FKBP12, accumulates in Alzheimer neurofibrillary tangles. Neurosci. Lett. 459 96–99 (2009). 19414059 10.1016/j.neulet.2009.04.062

[b16] Bowman G. R. & Geissler P. L. Equilibrium fluctuations of a single folded protein reveal a multitude of potential cryptic allosteric sites. Proc. Natl. Acad. Sci. U. S. A. 109, 11681–11686 (2012). 22753506 10.1073/pnas.1209309109 PMC3406870

[b17] Weber J. & Pande V. Characterization and rapid sampling of protein folding markov state model topologies. J. Chem. Theo. Comput. 7, 3405–3411 (2011). 10.1021/ct2004484 PMC3226725 22140370

[b18] Shirts M. & Pande V. S. Screen savers of the world unite. Science 290, 1903–1904 (2006). 17742054 10.1126/science.290.5498.1903

[b19] Burkhard P., Hommel U., Sanner M. & Walkinshaw M. D. The discovery of steroids and other novel FKBP inhibitors using a molecular docking program. J. Mol. Biol. 287, 853–858 (1999). 10222195 10.1006/jmbi.1999.2621

[b20] Weinan E. & Vanden-Eijnden E. Transition-path theory and path-finding algorithms for the study of rare events. Annu. Rev. Phys. Chem. 61, 391–420 (2010). 18999998 10.1146/annurev.physchem.040808.090412

[b21] Metzner P., Schütte C. & Vanden-Eijnden E. Transition path theory for markov jump processes. Multiscale Model Sim. 7, 1192–1219 (2009).

[b22] Mustafi S. M. *et al.* Structural basis of conformational transitions in the active site and 80's loop in the FK506-binding protein FKBP12. Biochem. J. 458, 525–536 (2014). 24405377 10.1042/BJ20131429 PMC3940039

[b23] Fischer S., Michnick S. & Karplus M. A mechanism for rotamase catalysis by the FK506 binding protein (FKBP). Biochemistry 32, 13830–13837 (1993). 7505615 10.1021/bi00213a011

[b24] Jain A. N. Surflex-Dock 2.1: robust performance from ligand energetic modeling, ring flexibility, and knowledge-based search. J. Comput.-Aided Mol. Des. 21, 281–306 (2007). 17387436 10.1007/s10822-007-9114-2

[b25] Rotonda J., Burbaum J. J., Chan H. K., Marcy A. I. & Becker J. W. Improved calcineurin inhibition by yeast FKBP12-drug complexes. Crystallographic and functional analysis. J. Biol. Chem. 268, 7607–7609 (1993). 7681823 10.2210/pdb1yat/pdb

[b26] Hess B., Kutzner C., van der Spoel D. & Lindahl E. Gromacs 4: Algorithms for highly efficient, load-balanced, and scalable molecular simulation. J. Chem. Theo. Comput. 4, 435–447 (2008). 10.1021/ct700301q 26620784

[b27] Lindorff-Larsen K. *et al.* Improved side-chain torsion potentials for the Amber ff99SB protein force field. Proteins: Struct., Funct., Bioinf. 78, 1950–1958 (2010). 10.1002/prot.22711 PMC2970904 20408171

[b28] Fujitani H. *et al.* Direct calculation of the binding free energies of FKBP ligands. J. Chem. Phys. 123, 084108 (2005). 16164283 10.1063/1.1999637

[b29] Wang J., Wolf R. M., Caldwell J. W., Kollman P. A. & Case D. A. Development and testing of a general amber force field. J. Comput. Chem. 25, 1157–1174 (2004). 15116359 10.1002/jcc.20035

[b30] Sousa da Silva A. & Vranken W. Acpype - antechamber python parser interface. BMC Research Notes 5, 367 (2012). 22824207 10.1186/1756-0500-5-367 PMC3461484

[b31] Jorgensen W., Chandrasekhar J., Madura J., Impey R. & Klein M. Comparison of simple potential functions for simulating liquid water. J. Chem. Phys. 79, 926 (1983).

[b32] Hess B., Bekker H., Berendsen H., Fraaije J. *et al.* Lincs: a linear constraint solver for molecular simulations. J. Comp. Chem. 18, 1463–1472 (1997).

[b33] Darden T., York D. & Pedersen L. Particle mesh ewald: An nlog (n) method for ewald sums in large systems. J. Chem. Phys. 98, 10089 (1993).

[b34] Hoover W. Canonical dynamics: Equilibrium phase-space distributions. Phys. Rev. A 31, 1695–1697 (1985). 9895674 10.1103/physreva.31.1695

[b35] Parrinello M. Polymorphic transitions in single crystals: A new molecular dynamics method. J. Appl. Phys. 52, 7182 (1981).

[b36] Beauchamp K. A. *et al.* MSMBuilder2: Modeling Conformational Dynamics at the Picosecond to Millisecond Scale. J. Chem. Theory Comput. 7, 3412–3419 (2011). 22125474 10.1021/ct200463m PMC3224091

[b37] Bowman G. R., Beauchamp K. A., Boxer G. & Pande V. S. Progress and challenges in the automated construction of markov state models for full protein systems. J. Chem. Phys. 131, 124101 (2009). 19791846 10.1063/1.3216567 PMC2766407

[b38] Chodera J. D., Singhal N., Pande V. S., Dill K. A. & Swope W. C. Automatic discovery of metastable states for the construction of Markov models of macromolecular conformational dynamics. J. Chem. Phys. 126, 155101 (2007). 17461665 10.1063/1.2714538

[b39] Prinz J.-H. *et al.* Markov models of molecular kinetics: Generation and validation. J. Chem. Phys. 134, 174105 (2011). 21548671 10.1063/1.3565032

[b40] Swope W. C., Pitera J. W. & Suits F. Describing protein folding kinetics by molecular dynamics simulations. 1. Theory. J. Phys. Chem. 108, 65716581 (2004).

[b41] Lapelosa M., Gallicchio E. & Levy R. M. Conformational Transitions and Convergence of Absolute Binding Free Energy Calculations. J. Chem. Theory Comput. 111215080904009 (2011). 10.1021/ct200684b PMC3285237 22368530

[b42] Humphrey W., Dalke A. & Schulten K. Vmd: visual molecular dynamics. J. Mol. Graph. 14, 33–38 (1996). 8744570 10.1016/0263-7855(96)00018-5

[b43] Holt D. A. *et al.* Design, synthesis, and kinetic evaluation of high-affinity FKBP ligands and the X-ray crystal structures of their complexes with FKBP12. J. Am. Chem. Soc. 115, 9925–9938 (1993).

[b44] Adams P. D. *et al.* PHENIX: a comprehensive Python-based system for macromolecular structure solution. Acta Crystallogr. D Biol. Crystallogr. 66, 213–221 (2010). 20124702 10.1107/S0907444909052925 PMC2815670

