# Supplementary material for: Cloud computing approaches for prediction of ligand binding poses and pathways
Source: Sci Rep. 2015 Jan 22;5:7918. doi: 10.1038/srep07918 (PMC4302315; doi:10.1038/srep07918)

Supplementary information for cloud computing approaches for prediction of ligand binding poses and pathways

Morgan Lawrenz[1], Diwakar Shukla[1,2] & Vijay S. Pande[1,2]

[1]*Department of Chemistry, Stanford University, Stanford, CA 94305*
[2]*SIMBIOS NIH Center for Biomedical Computation, Stanford University, Stanford, CA 94305*

Supplementary Table 1. Adaptive sampling scheme for FKBP12 ligand binding simulations.

	Round 1 (μs)	Round 2	Round 3	Total (μs)	Bind events	Unbind events
L2	3.7	107.2	69.8	180.7	883[a]	222[b]
L3	3.3	126.9	70.1	200.3	891	273
L6	3.0	115.4	71.1	189.5	560	185
L9	3.1	128.2	73.2	204.5	494	141
AND	73.4	76.4	-	149.6	411	120

[a]Bind criteria: In trajectory, W59 sidechain - ligand nitrogen atom (oxygen for Androstan) distance transitions to $>$ 1.5 Åwhen cumulative average is $<$ 6 Å.
[b]Unbind criteria is reverse of bind.

Supplementary Table 2. Final ranking of high probability MSM states for FKBP12 ligands. Four states with highest equilibrium probabilities from the final MSM are listed with the state rmsd to the reference bound state. Only L9 has a deposited crystal structure; other ligand poses are derived from the FK506 structure 1YAT as described in the main text. The Androstan reference structure is the final predicted pose.

pose	AND		L2		L3		L6		L9	
	π^a	rmsd[b]	π	rmsd	π	rmsd	π	rmsd	π	rmsd
0	0.2	0.0	0.12	1.3	0.23	1.2	0.04	2.6	0.11	0.7
1	0.15	6.0	0.06	6.2	0.05	5.3	0.03	7.7	0.10	6.6
2	0.06	1.3	0.04	5.9	0.03	2.0	0.03	6.8	0.09	4.8
3	0.04	6.1	0.03	5.6	0.03	4.9	0.02	4.2	0.05	5.3

[a]Normalized equilibrium population from the MSM.

[b]Root mean square deviation (Å) from reference (experimental or predicted) pose.

Supplementary Table 3. Comparison of computed and experimental FKBP ligand association rates. MSM-computed association rates for the ligands in this study (Holt, *et al.*) are listed, but as no experimental association rates are available for these particular ligands, we compare to related FK506-derived ligands (Tassa, *et al.*) with a similar binding affinity range and association rates from surface plasmon resonance (SPR). The strongest binders from both ligand series have rates that compare well at $\approx 20 \cdot 10^6 M^{-1} s^{-1}$.

	Tassa, et al. Ligands (exp)	Holt, et al. Ligands (calc)
K_{on} ($10^6 M^{-1} s^{-1}$)	0.019 - 230	19 - 227

Supplementary Table 4. Top flux pathway descriptions for FKBP12 ligands. For each pathway, a flux for each ligand is shown as a percentage out of the total flux represented by the analyzed top flux pathways. A pathway was analyzed if it met the criterion of having $> 50\%$ flux of the maximum flux pathway. The encounter complex is defined as the first observed on-pathway metastable state with formed ligand-protein interactions and is described with a summary of the interactions formed: near bound, within 5 Å of the predicted bound state; flipped intermediate, within the active site but flipped, as the example described in Supplementary Fig. 5; 80's loop interaction, ligand binding either on the C-terminal or N-terminal ends of the loop formed by residues 84-91; nonspecific site, ligand interactions with sites A, B, or C as labeled in Supplementary Fig. 4.

encounter complex	AND	L2	L3	L6	L9
near-bound	5	13	32	0	0
flipped intermediate	0	53	15	5	36
80's Loop interaction	25	24	0	71	36
nonspecific site	69	10	53	24	28

Supplementary Table 5. List of lag times used for all MSMs in convergence plots. MSMs at listed aggregate simulation times were built using the lag time based on the implied timescale convergence. Models that did not demonstrate converged time scales were discarded and are not listed.

L2		L3		L6		L9		AND	
time[a]	lag[b]	time	lag	time	lag	time	lag	time	lag
0.6	0.6	0.5	0.4	1.0	0.4	0.5	0.4	5.6	0.4
6.0	6.0	3.3	2.0	6.6	1.0	0.7	0.4	21.5	0.4
11.3	6.0	22.9	1.0	16.	1.0	1.4	0.6	41.7	0.4
16.6	6.0	41.6	1.0	31.	3.0	6.1	3.0	51.5	0.4
20.7	1.0	58.3	2.0	47.2	1.0	23.2	0.8	69.3	0.4
38.3	1.0	73.4	3.0	60.1	2.0	33.4	0.8	77.1	0.4
54.2	1.0	93.3	4.0	71.1	2.0	42.9	0.8	90.1	2.0
68.4	2.0	116.6	6.0	84.6	12.0	52.0	0.8	105.0	2.0
80.7	2.0	127.0	6.0	84.6	12.0	60.5	2.0	113.0	2.0
92.0	4.0	136.5	2.0	96.3	5.0	76.2	1.5	130.9	2.0
102.4	2.0	153.2	6.0	107.2	10.0	103.4	6.0	142.3	2.0
116.4	4.0	166.9	10.0	113.9	10.0	120.4	6.0	149.6	2.0
128.7	10.0	178.0	12.0	125.0	10.0	130.7	3.0		
139.5	6.0	184.4	10.0	151.4	12.0	140.2	3.0		
151.4	6.0	196.0	8.0	163.5	10.0	156.9	2.0		
163.0	6.0	200.3	8.0	174.5	8.0	176.7	5.0		
173.1	6.0	200.3	8.0	189.5	10.0	204.5	10.0		
180.7	10.0								

[a]Aggregate simulation time in μs. [b] Lag time used for model in ns.

Supplementary Figure 1. Convergence of lowest free energy ligand MSM state. The lowest free energy state was selected from MSMs built at $\approx 10\ \mu s$ intervals as aggregate sampling time is increased. We plot the state RMSD to the reference poses derived from crystallography (described in the main text), or in the case of Androstan the final predicted pose, as the rolling mean with standard deviations over 2 data points ($\approx\ 20\ \mu s$). Data shown for ligands L2 **(a)**, L3 **(b)**, L6 **(c)**, L9 **(d)**, Androstan **(e)**. Adaptive sampling times as described in Supplementary Table 1 are marked with red dashed lines. Nonspecific sites A, B, and C are labeled for L2 as non-converged binding sites and described in Supplementary Fig. 4.

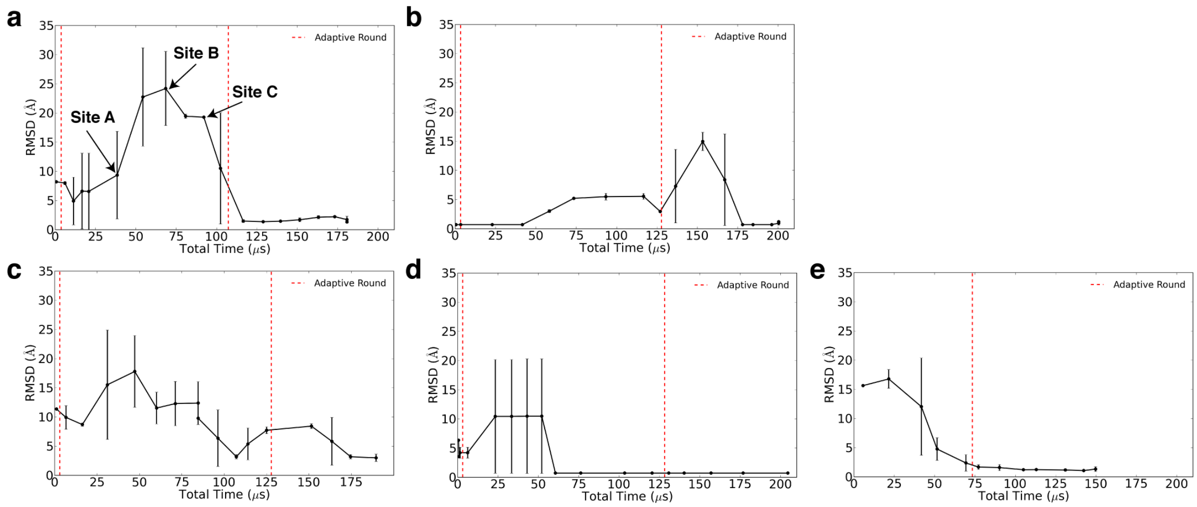

Supplementary Figure 2. Predicted FKBP ligand binding poses from MSM free energies. **(a)** Overlap of the MSM-predicted binding poses of FK506-derived ligands (shown in stick representation) with the electron density derived for FK506 (PDBID 1YAT). For clarity, the densities shown are restricted to FK506 atoms which correspond to the derived ligand. **(b)** Free energy isosurfaces at 1.0 kcal/mol of the 3-D MSM-weighted free energy map are shown for all ligands.

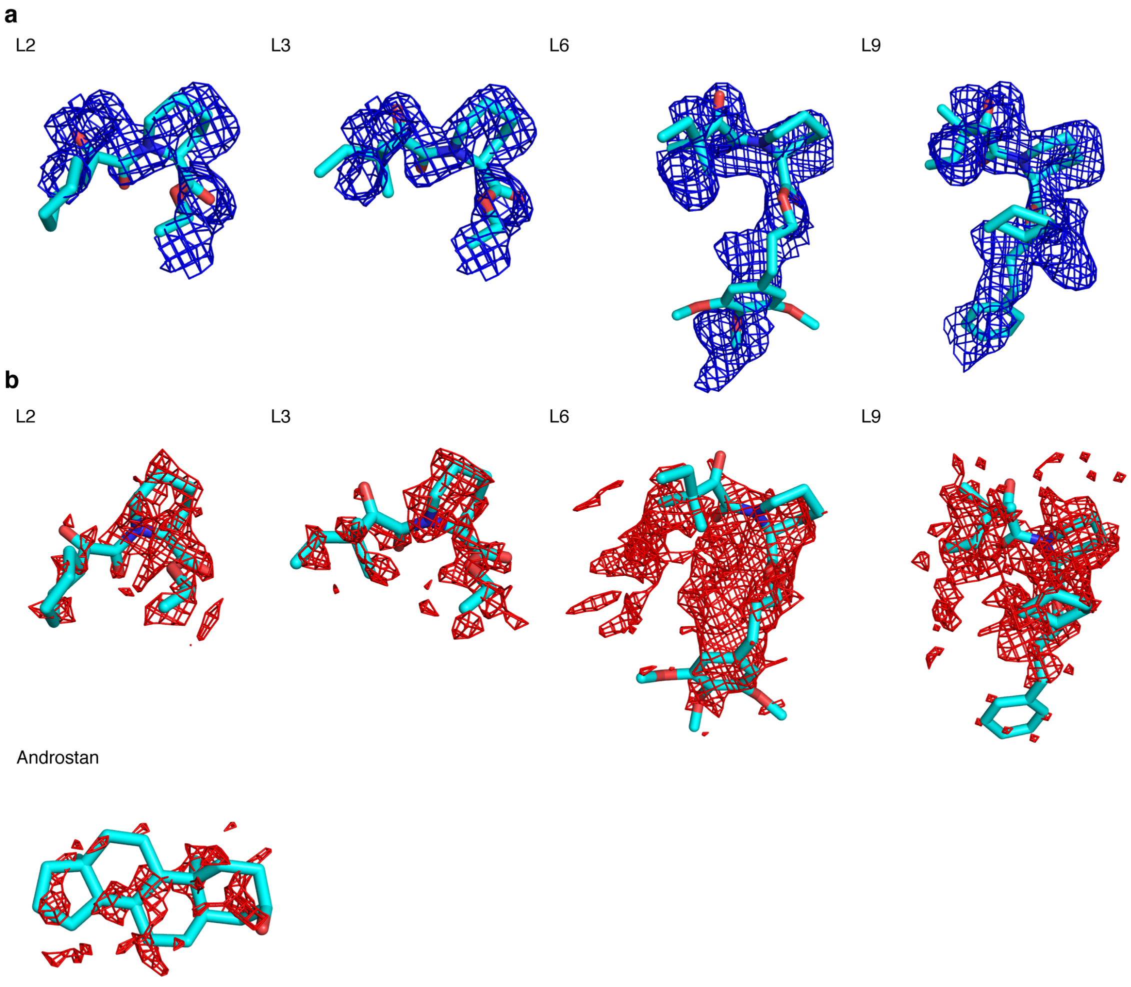

Supplementary Figure 3. Low free energy surfaces can predict druggable regions of the binding site. **(a)** The MSM free energy isosurface at 1.5 kcal/mol is shown for L2 (in cyan stick representation), which extends down the N terminal region of the 80s loop (green square) **(b)** L6 (shown in cyan stick representation), as well as L9, and FK506 (not shown) make contacts in this same region, which contribute to an approximately two orders of magnitude increase in binding affinity.

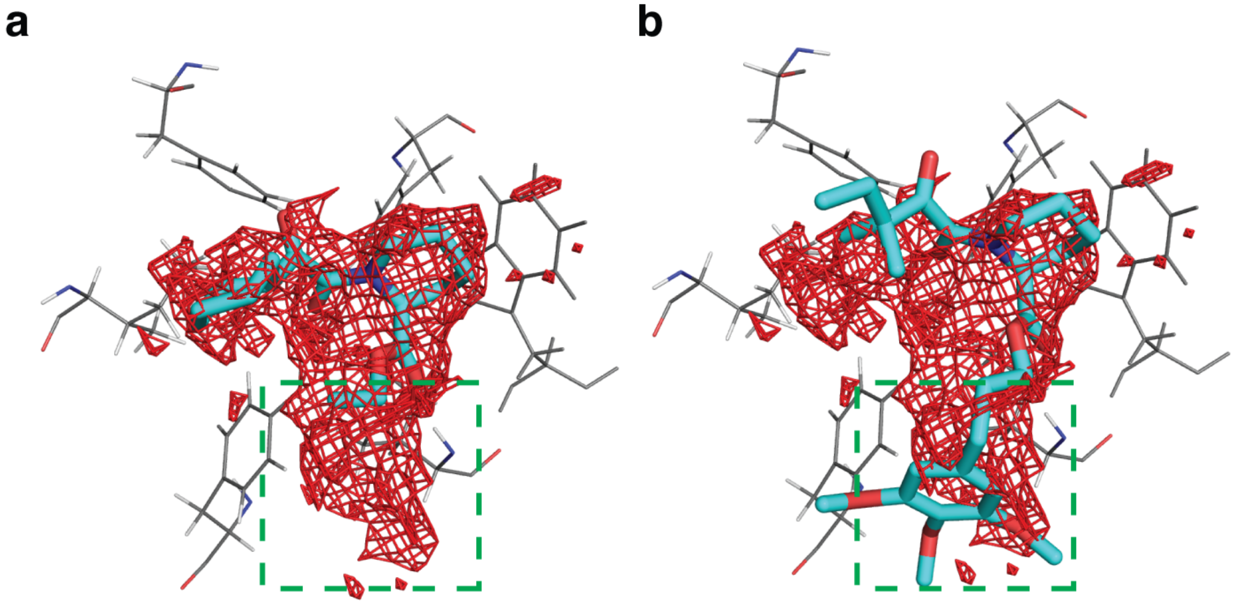

Supplementary Figure 4. Predicted nonspecific binding sites. The MSM-weighted free energy map at the isosurface of 3.0 kcal/mol reveals high free energy binding sites on the protein that are common between all ligands. The primarily hydrophobic interactions formed with the ligand in the three sites, labeled A, B, and C, are also depicted

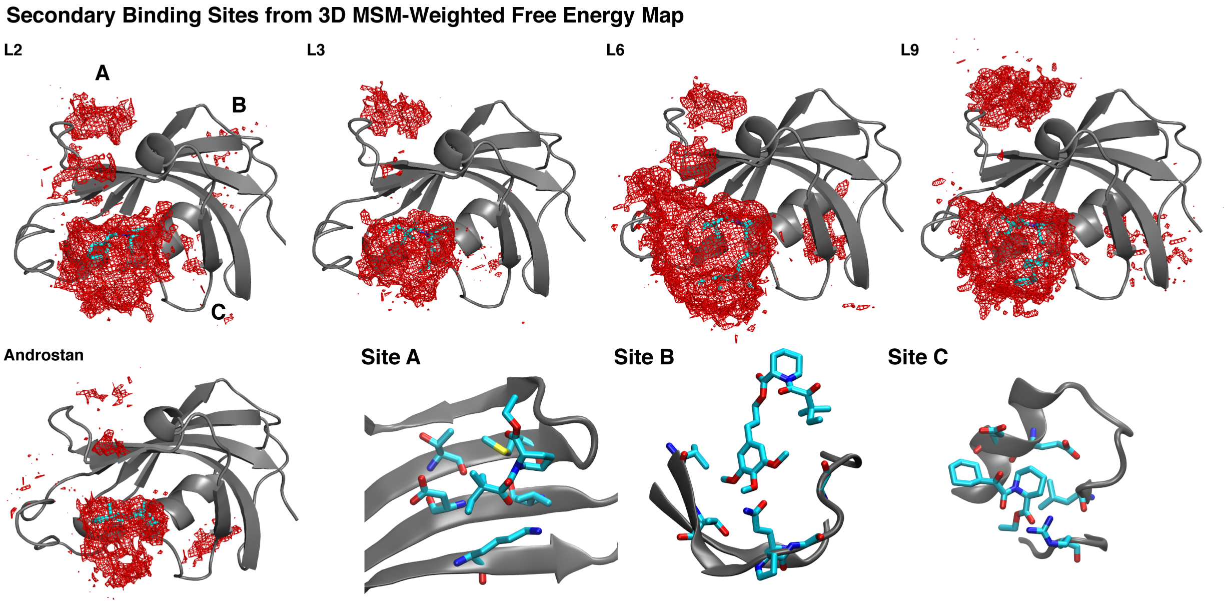

Supplementary Figure 5. Hydrogen bond shuttling mechanism in common FK506 ligand and intermediate. The equilibrium population for these states are L2: 0.8%, L3: 0.8%, LG6: 0.5%, LG9: 0.9%. **(a)** The structure of the intermediate is shown for L6 and L2, with a comparison to the predicted crystal binding mode for L2. In the intermediate, the ligand isoleucine-mimetic or ring sidechain (represented by distances to the central atom c3) forms hydrophobic interactions with residues F99 and W59, but switches to interactions with F36 and I90. This switching is shown in the distance distributions in **(b)**. Shown in **(c)** and **(d)**, the ligand carbonyl groups, labeled o1, o2, and o3, initiate hydrogen bonds in the intermediate with both the Y82 hydroxyl and I56 backbone (red distributions). Conversion to the bound state stabilizes the o2 interaction with Y82 and allows o3 to find its hydrogen bond with I56.

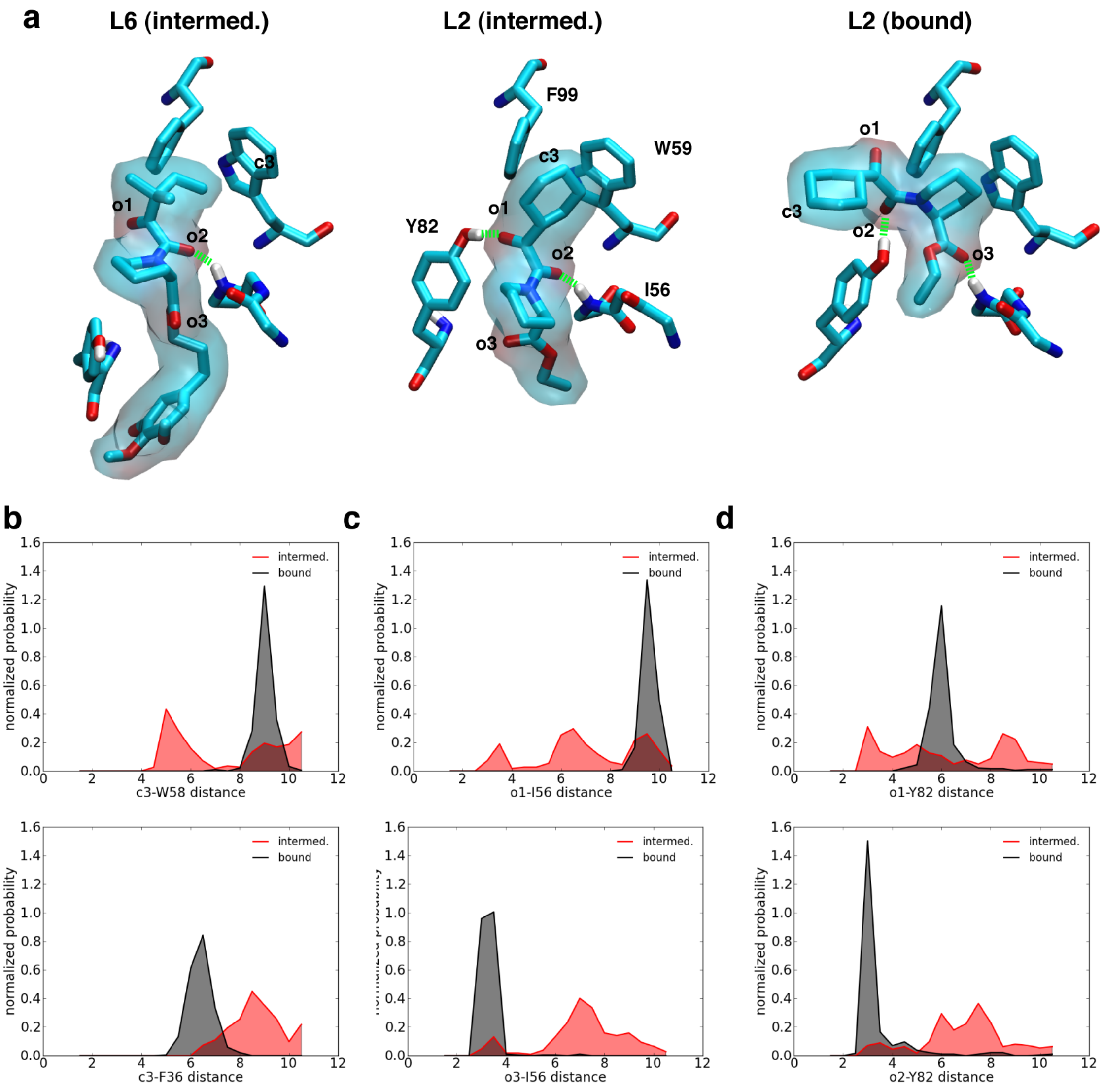

Supplementary Figure 6. Implied timescales for the final MSM for all ligands. Converging timescales, or the log of the eigenvalues μ computed from the transition matrix P_{ij} for models constructed at the final aggregate sampling time. Various lag times τ are shown with separate colors for each timescale for L2 **(a)**, L3 **(b)**, L6 **(c)**, L9 **(d)**, Androstan **(e)**. The lag time of 10 ns was chosen for final analysis, shown for all plots as a dashed black line in the initial plateau region. The appropriate lag time for an MSM in practice requires a balance of demonstrated Markovian behavior (see Methods in the main text) with maintenance of good statistics that contribute to the matrix *Cij*. Here the smallest lag time was chosen in regions where convergence behavior is first demonstrated.

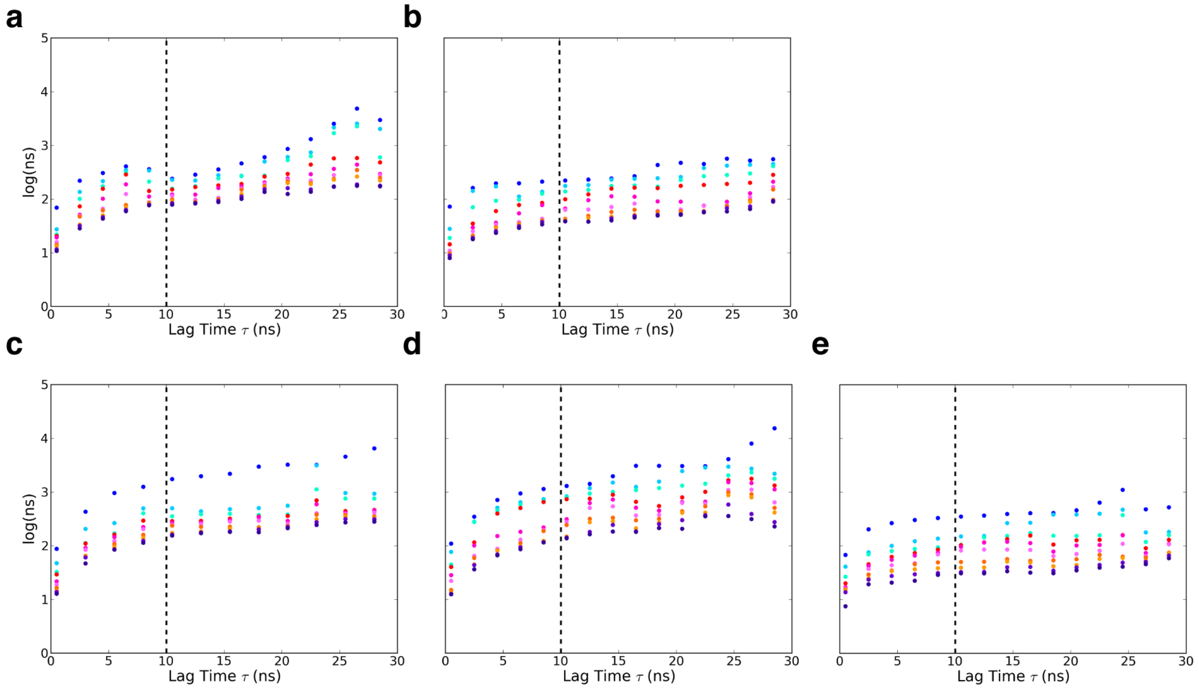

Supplement: Supplementary Information — for cloud computing approaches for prediction of ligand binding poses and pathways [file srep07918-s1.pdf]
